# Purification, characterization, and antioxidant ability of polysaccharides from *Phascolosoma esculentas*

**DOI:** 10.1002/fsn3.3961

**Published:** 2024-01-12

**Authors:** Fengfang Zhou, Binxin Cai, Shaojiang Ruan, Qi Wei

**Affiliations:** ^1^ College of Life Sciences Ningde Normal University Ningde China; ^2^ Engineering Research Center of Mindong Aquatic Product Deep Processing Fujian Province University Ningde China; ^3^ State Oceanic Administration Hercynian Special Biological Germplasm Resources and Biological Product Development Public Service Platform Ningde China

**Keywords:** antioxidant, characterization, *Phascolosoma esculenta*, polysaccharides

## Abstract

The polysaccharide was extracted from *Phascolosoma esculenta* (PEP). Two purified polysaccharides (PEP‐1 and PEP‐2) were obtained by the column chromatography separation method. The molecular weights of PEP‐1 and PEP‐2 were 33.6 and 5.7 × 10^3^ kDa, respectively. PEP‐1 and PEP‐2 had the same monosaccharides composition, but their molar ratios varied. The in vitro antioxidant activity of the PEP, PEP‐1, and PEP‐2 were investigated by scavenging free radicals like 3‐ethylbenzoth‐iazoline‐6‐sulfonic acid (ABTS), •OH, and 2,2‐diphenyl‐1‐picrylhydrazyl (DPPH). Additionally, the in vivo antioxidant activity of PEP‐1 was examined using the *Caenorhabditis elegans* (*C. elegans*) organism. Results showed that PEP‐1 was much more effective than PEP and PEP‐2 at scavenging DPPH, •OH, and ABTS radicals. Additionally, PEP‐1 strengthened *C. elegans'* ability to endure oxidative stress. PEP‐1 possessed the in vivo antioxidant capacity, including the reactive oxygen species (ROS) content reducing, and protective effect on antioxidant enzyme activities in *C. elegans*. In summary, PEP, PEP‐1, and PEP‐2 might have the potential to develop as functional foods and clinical medications.

## Graphical abstract

The polysaccharide was extracted from *Phascolosoma esculenta*. The characterization of polysaccharides were investigated. The polysaccharide from *Phascolosoma esculenta* possessed in vitro and in vivo antioxidant activities.

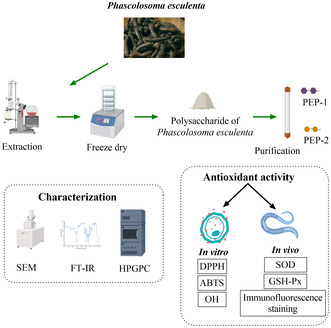

## 1 INTRODUCTION

A physiological state known as oxidative stress occurs when the generation of reactive oxygen species (ROS) and antioxidant defense systems are out of balance. An excessive amount of ROS can lead to cellular malfunction and structural damage. Oxidative stress is involved with many diseases, including cancer, hyperglycemia, and hypertension (Ebraheem et al., 2023). Therefore, the further development of natural antioxidants is essential to the food and pharmaceutical industry. Marine organisms contain a lot of nutrients and active substances. Such as active peptides, amino acids, and polysaccharides. Marine polysaccharides are obtained from a variety of sources, including marine animals, seaweed, and marine microbial. The polysaccharides from marine resources possess obvious therapeutic effects against many diseases (Liu et al., 2020; Sharma et al., 2023; Yang et al., 2020). Ju et al. (2023) showed that marine polysaccharides have effective effects against tumors by inhibiting tumor angiogenesis and activating the immune system. Polysaccharides from marine macroalga *Hydropuntia edulis* had a beneficial effect against α‐amylase and α‐glucosidase activities and had potential therapeutic lead against hyperglycemia (Antony et al., 2022). Many studies revealed that marine polysaccharides possess antioxidant activity (Mou et al., 2018; Qin et al., 2018).

*Phascolosoma esculenta* (*P. esculenta*), a member of the *Phascolosoma* genus, is an important food resource in China (Wu et al., 2020). *P. esculenta* is also known as “jelly seaworm,” which contains mostly collagen. Thus, it can keep its beauty and health. The most essential bioactive component of *P. esculenta* is polysaccharides. Recently, several researchers extracted polysaccharides from *P. esculenta* and discovered that the polysaccharides of *P. esculenta* exhibited antioxidant properties (Liang, 2008; Wu et al., 2020). However, there were limited researches on the antioxidant capacity and structural study of the purified polysaccharides from *P. esculenta*. Much more attention is required for the development and extension of *P. esculenta* polysaccharide applications.

In this study, two purified polysaccharides were obtained from *P. esculenta*. The characterization of the polysaccharides was tested. Additionally, the polysaccharides' antioxidant properties were evaluated. This research would contribute to the development of polysaccharides from *P. esculenta* as a functional food and natural drug source that can prevent and treat chronic diseases.

## 2 MATERIALS AND METHODS

### 2.1 Materials and chemicals

*Phascolosoma esulenta* was bought from Ningde (Fujian, China), vacuum‐packed, and stored at −20°C. 2,2‐diphenyl‐1‐picrylhydrazyl (DPPH), papain, 2,7‐dichloro‐dihydro fluorescein diacetate (DCFH‐DA), 3‐ethylbenzoth‐iazoline‐6‐sulfonic acid (ABTS), and ascorbic acid (Vc) were acquired from Aladdin Biotechnology Co., Ltd. (Shanghai, China). Q Beads 6FF was acquired from Smart‐Lifesciences Biotechnology Co., Ltd. (Changzhou, China). Malondialdehyde (MDA), superoxide dismutase (SOD), and Glutathione peroxidase (GSH‐Px) kits were brought from Nanjing Jiancheng Biotechnology Co., Ltd. (Nanjing, China). Wild‐type *Caenorhabditis elegans* (*C. elegans*) and uracil leakage defective *Escherichia coil* OP50 (OP50) were acquired from SunyBiotech Co., Ltd. (Fuzhou, China).

### 2.2 Extraction and purification

Fresh *P. esculenta* was washed, and combined with sodium acetate buffer (0.1 M, 1:30 w/v), and then homogenized. After that, the homogenate was mixed with 5% papain. The mixture was stirred and incubated in a water bath (DK‐600 Shanghai Feiyue Instrument Co., Ltd., Shanghai, China) at 55°C for 6 h. After the incubation, the mixture was kept at 95°C for 10 min. Afterward, the mixture was centrifuged at 4°C, 7606 *g* for 25 min. The supernatant was combined with sevag reagent (n‐butanol: chloroform = 1:4, v/v) to remove the protein. The extract was mixed with 95% ethanol in a volume ratio of 1:3 (v/v) and then kept in a refrigerator (4°C, 12 h). The polysaccharides of *P. esculenta* (PEP) were obtained after freeze‐drying (FDU‐2110, Eyela Co., Ltd., Tokyo Japan).

PEP (5 mg/mL) was combined with distilled water and purified using Q Beads 6FF column (2.6 × 60 cm). Distilled water and a gradient NaCl solution (0.1–0.5 mol/mL) were used to elute the column. The flow rate was 3 mL/min. Each tube has a capacity of 10 mL. The polysaccharide content was analyzed as reported by Yang et al. (2020). The eluate was then dialyzed (3500 Da) and freeze‐dried to produce *P. esculenta* purified polysaccharides (PEP‐1 and PEP‐2). Using the following formula, the extraction yield of polysaccharides was determined.

$$\text{Extraction yield}\;\left(\%\right)=\frac{A_{i}}{A_{0}}\times 100\%$$

*A*_i_: the weight of the dried polysaccharides; *A*_0_: the weight of dried *P. esculenta*.

### 2.3 Molecular weight and monosaccharide composition analysis

The molecular weights of the PEP‐1 and PEP‐2 were determined using high‐performance gel permeation chromatography (HPGPC, 1260 Infinity MDS, Agilent, USA) with a deferential refraction detector and a multi‐angle laser light scatterer (Guo et al., 2020). The content of monosaccharide was analyzed by high‐performance liquid chromatography (1200, Agilent, USA) as reported by Liu et al. (2020).

### 2.4 Fourier transform infrared (FT‐IR) spectroscopy analysis

Potassium bromide (100 mg) was combined with polysaccharides (1 mg) before being formed into a disk. The FT‐IR spectral analysis was performed with a Fourier transform infrared spectrophotometer (Nicolet IS50, Thermo Fisher, USA) in the 4000 to 400/cm range.

### 2.5 Scanning electron microscopy (SEM) analysis

Scanning electron microscopy (JSM‐840, JEOL, Japan) was used to study the microstructure of PEP‐1 and PEP‐2.

### 2.6 In vitro antioxidant activity assays

#### 2.6.1 DPPH radical scavenging activity

DPPH radicals scavenge activity was determined using the procedure of Wei et al. (2022). A volume of 100 μL of polysaccharides (0.2, 0.4, 0.6, 0.8, 1.0 mg/mL) was combined with DPPH solution (100 μL, 0.1 mmol/L). Additionally, the 96‐well plate was placed in an incubator at 37°C for 30 min. At 517 nm, the absorbance was measured. Using the following formula, the DPPH scavenging activity was determined.

$$\text{DPPH scavenging activity}\;\left(\%\right)=\left(1‐\frac{A_{i}‐A_{j}}{A_{0}}\right)\times 100\%$$

*A*_*i*_ is the absorbance of DPPH solution with the sample solution, *A*_0_ is the absorbance of DPPH solution without the sample solution, and *A*_*j*_ is the absorbance of the sample solution.

#### 2.6.2 Hydroxyl radical (•OH) scavenging activity

The •OH scavenging activity was determined as reported by Deng et al. (2021). Subsequently, 2 mL of polysaccharides (0.5, 1.0, 1.5, 2.0, 2.5 mg/mL), salicylic acid‐ethanol solution (2 mL, 9 mmol/L), hydrogen peroxide solution (2 mL, 30% v/v), and FeSO_4_ solution (2 mL, 9 mmol/L) were successively transferred into a centrifuge tube, vortexed immediately and then reacted at 37°C for 30 min. At 510 nm, the absorbance was measured. Using the following formula, the •OH scavenging activity was determined.

$$•\mathrm{OH}\;\text{scavenging activity}\;\left(\%\right)=\left(1‐\frac{A_{i}‐A_{j}}{A_{0}}\right)\times 100\%$$

*A*_*i*_ is the sample absorbance, *A*_0_ and *A*_*j*_ are the distilled water absorbance instead of sample or hydrogen peroxide, respectively.

#### 2.6.3 ABTS radical scavenging activity

The ABTS radical scavenging activity was assayed as described by Wei et al. (2022). Simply, the same volume of potassium persulfate solution (5 mL, 2.45 mM) and ABTS solution (5 mL, 7 mM) were combined to start the reaction for 12 h at 25°C. At 734 nm, the absorbance of the mixture was adjusted to 0.7 ± 0.05, using distilled water. Subsequently, A volume of 100 μL of polysaccharides (0.5, 1.0, 1.5, 2.0, 2.5 mg/mL) was combined with 100 μL of the mixture solution. Additionally, the 96‐well plate was placed in an incubator at 25°C for 6 min. At 734 nm, the absorbance was measured. Using the following formula, the ABTS scavenging activity was determined.

$$\text{ABTS scavenging activity}\;\left(\%\right)=\left(1‐\frac{A_{i}‐A_{j}}{A_{0}}\right)\times 100\%$$

*A*_*i*_ is the absorbance of ABTS solution with the sample solution, *A*_0_ is the absorbance of ABTS solution without sample solution, and *A*_*j*_ is the absorbance of the sample solution.

### 2.7 In vivo antioxidant activity assays

#### 2.7.1 *Caenorhabditis elegans* and culture conditions

*Caenorhabditis elegans* was fed *E. coli* OP50as the food source. Nematode growth medium (NGM) plates were used to cultivate the *C. elegans*. The incubation temperature for *C. elegans* was 20°C.

#### 2.7.2 Oxidative stress resistance assay

The oxidative stress resistance assay was determined as reported by Mekheimer et al. (2012). Briefly, the synchronized L4 of *C. elegans* were treated with PEP‐1 (0, 0.25, 0.5, 1 mg/mL) and cultured in NGM agar plates (20°C, 4 days). The control group was treated with 0 mg/mL of PEP‐1. Subsequently, the *C. elegans* were maintained in NGM agar plates containing 0.3% H_2_O_2_, then stored at 20°C. Every hour, the mortality rate of *C. elegans* was recorded.

#### 2.7.3 Determination of the ROS level

ROS levels were determined using 2′,7′‐dichlorofluorescein diacetate (DCFH‐DA) as reported by Lin et al. (2023). The synchronized L4 of *C. elegans* were treated with PEP‐1 (0, 0.25, 0.5, 1 mg/mL) and cultured in NGM agar plates (20°C, 4 days). Thereafter, *C. elegans* were mixed with DCFH‐DA (20 μM) and then reacted for 2 h without light. Subsequently, *C. elegans* were transferred to a glass slide and photographed by fluorescence microscope (DM6B, Leica, Germany). Additionally, the relative fluorescence intensity was measured by an enzyme‐labeled instrument (Feyond‐A300, Shanghai, China).

#### 2.7.4 Oil red O (ORO) staining assay

ORO staining was determined as reported by Lin et al. (2019). The synchronized L4 of *C. elegans* were treated with PEP‐1 (0, 0.25, 0.5, 1 mg/mL) and cultured in NGM agar plates (20°C, 4 days). 1,2‐propanediol was used to dehydrate *C. elegans* for 5 min. Subsequently, *C. elegans* were incubated with ORO solution at 20°C for 24 h. *C. elegans* were treated with phosphate buffer saline and 1,2‐propanediol after incubation. *C. elegans* were transferred to 2% agarose slide. The morphology was observed by a DMC2900 microscope (Leica, Germany).

#### 2.7.5 Antioxidant enzyme activities and MDA content

The synchronized L4 of *C. elegans* were treated with PEP‐1 (0, 0.25, 0.5, 1 mg/mL) and cultured in NGM agar plates (20°C, 4 days). *C. elegans* were treated with phosphate buffer saline, and then homogenized with an ice‐cold tissue grinder. The bicinchoninic acid (BCA), GSH‐Px, MDA, and SOD were determined separately based on the kit instructions (Nanjing Jiancheng Biotechnology Co., Ltd., Nanjing, China).

#### 2.7.6 Statistical analysis

GraphPad Prism 10 was used to process the images. Differences were considered significant at *p* < .05. All data were expressed as mean ± standard deviation of three replicates.

## 3 RESULTS

### 3.1 Yield

**FIGURE 1:**
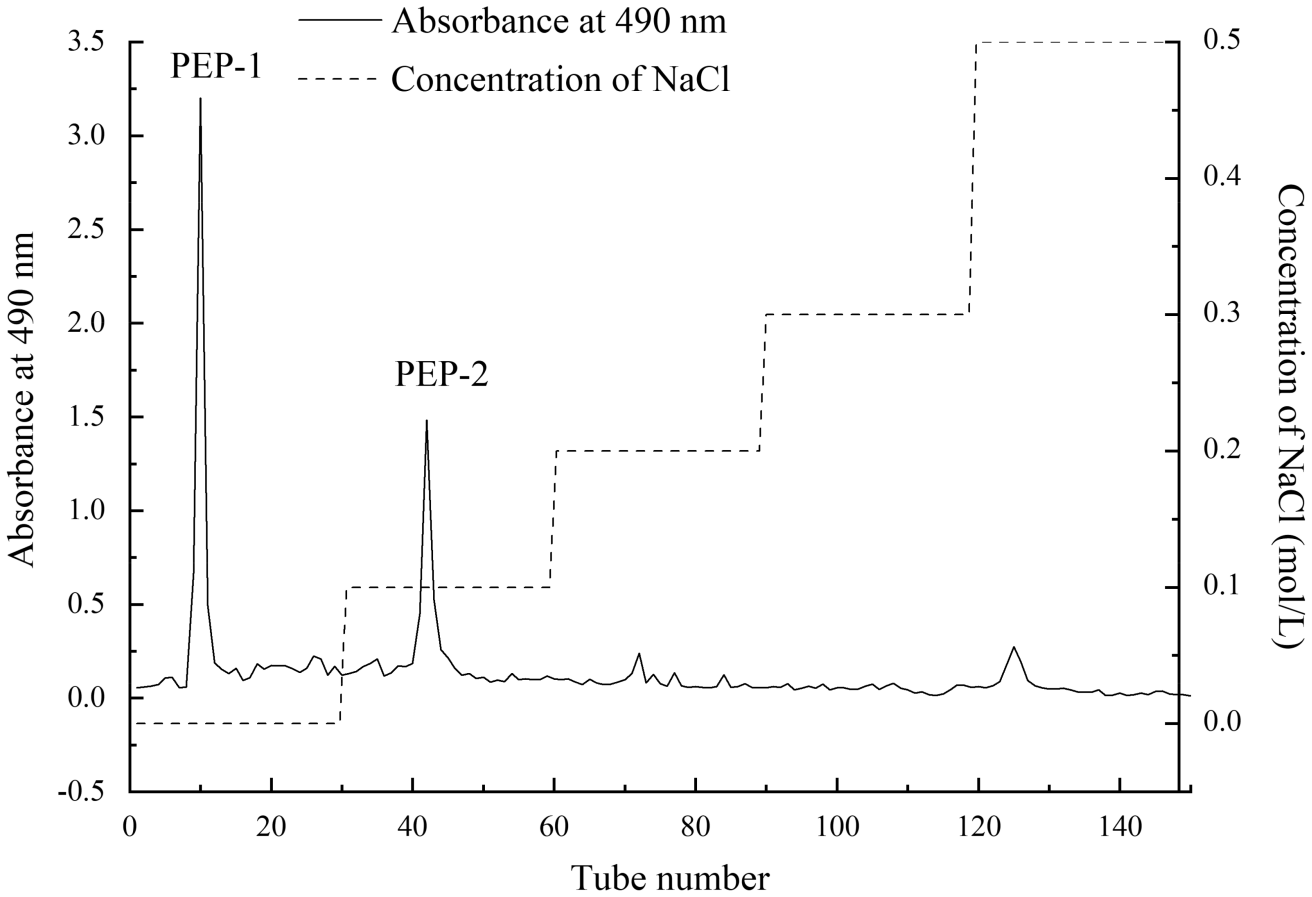
Elution curve of polysaccharides from *Phascolosoma esculenta* on Q Beads 6FF column chromatography.

PEP was obtained from *P. esculenta*, resulting in a yield of 7.91%. As shown in Figure 1, the PEP was separated by Q Beads 6FF column and the purified polysaccharides of *P. esculenta* (PEP‐1 and PEP‐2) were obtained. PEP‐1 eluted by distilled water with a yield of 3.75%. PEP‐2 eluted by 0.1 mol/L NaCl with a yield of 2.12%.

### 3.2 Molecular weight and the analysis of monosaccharide composition

**FIGURE 2:**
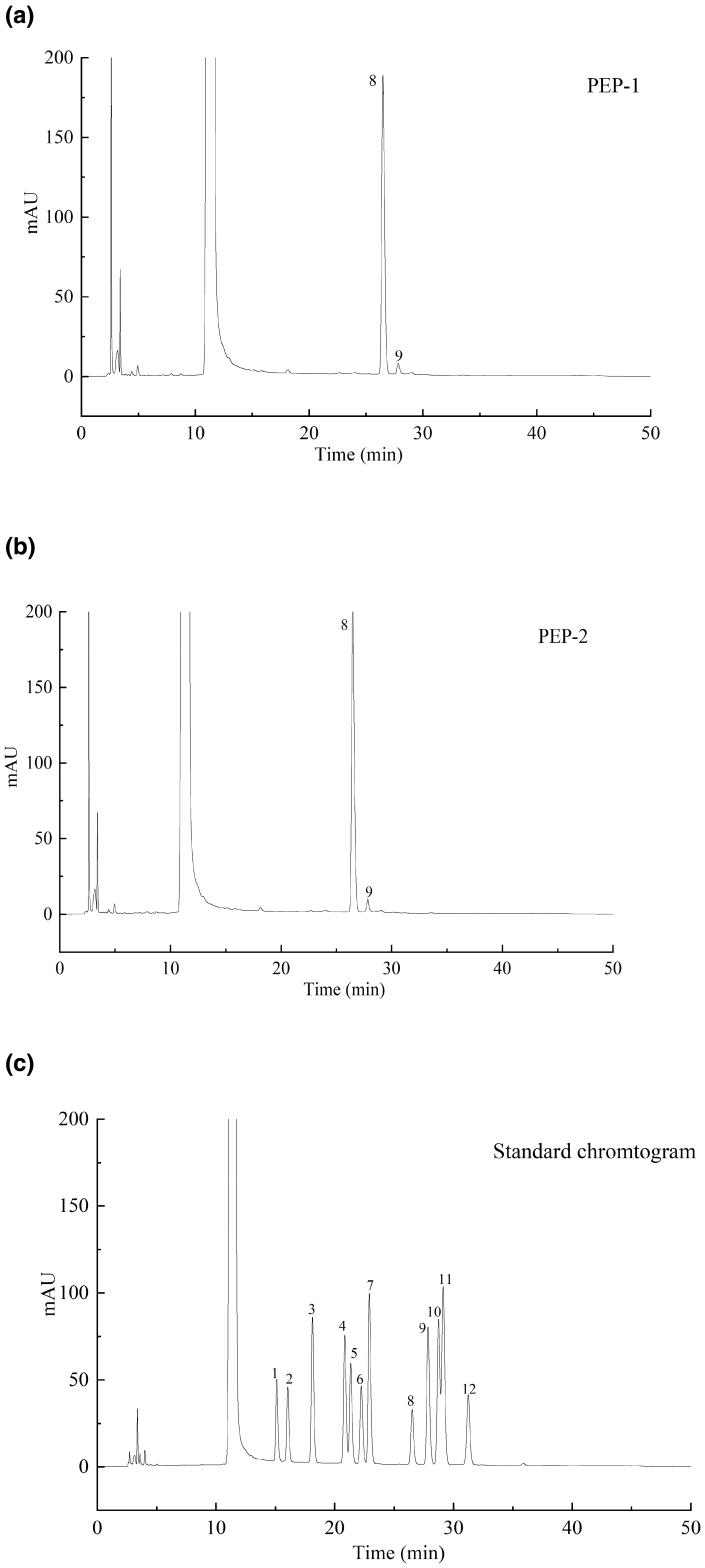
HPLC analysis for the monosaccharide composition of the purified polysaccharides from *Phascolosoma esculenta*. (a) PEP‐1, (b) PEP‐2, and (c) standard monosaccharides. The number indicated the corresponding monosaccharide. 1, Guluronic acid; 2, Mannuronic acid; 3, Mannose; 4, Ribose; 5, Rhamnose; 6, Glucuronic acid; 7, Galacturonic acid; 8, Glucose; 9, Galactose;10, Xylose; 11, Arabinose; and 12, Fucose.

The results of molecular weight measurement for PEP‐1 and PEP‐2 were depicted in Table 1. The molecular weight of PEP‐1 and PEP‐2 was 33.6 and 5.7 × 10^3^ kDa, respectively. As shown in Figure 2, ribose, rhamnose, glucose, mannuronic acid, fucose, xylose, galactose, glucuronic acid, arabinose, guluronic acid, mannose, and galacturonic acid were found in PEP‐1 and PEP‐2. The glucose was the primary monosaccharide component in PEP‐1 and PEP‐2, constituting 94.57% and 96.79%, respectively, followed by galactose, accounting for 3.39% and 1.61%, respectively.

**TABLE 1:** Molecular weight and monosaccharide compositions of purified polysaccharides from *Phascolosoma esculenta* (PEP‐1 and PEP‐2).

	PEP‐1	PEP‐2
Mw (kDa)	33.6	5.7 × 10^3^
Sugar (%)		
Guluronic acid	0.06	0.13
Mannuronic acid	0.15	0.17
Mannose	0.81	0.56
Ribose	0.04	0.03
Rhamnose	0.12	0.09
Glucuronic acid	0.13	0.09
Galacturonic acid	0.00	0.00
Glucose	94.59	96.79
Galactose	3.39	1.62
Xylose	0.11	0.10
Arabinose	0.34	0.21
Fucose	0.04	0.06

### 3.3 FT‐IR spectrum analysis

**FIGURE 3:**
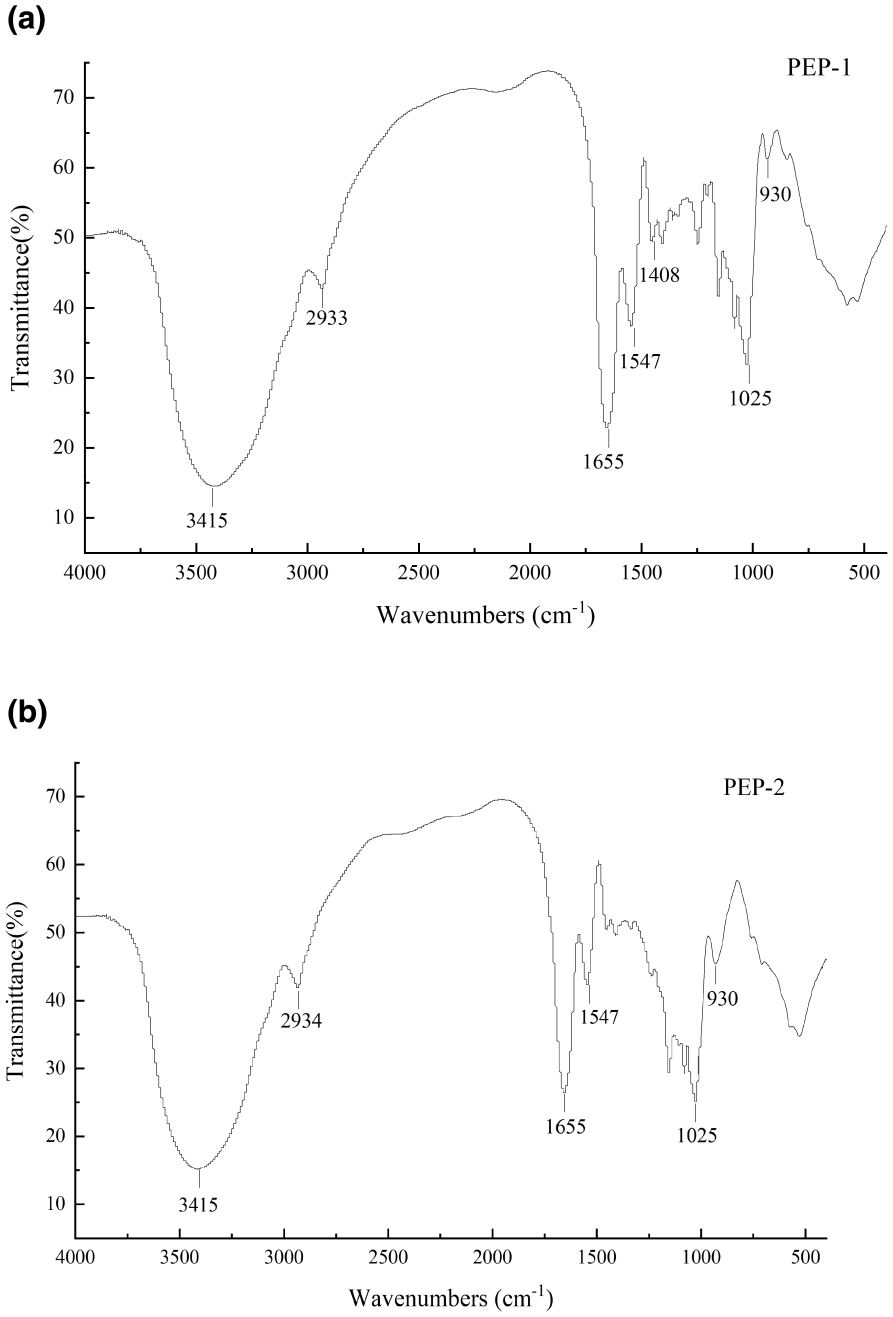
FT‐IR spectrum of the purified polysaccharides from *Phascolosoma esculenta*. (a) PEP‐1, and (b) PEP‐2.

The chemical bonds of PEP‐1 and PEP‐2 were investigated by FT‐IR (Figure 3). The broad band at 3415/cm was attributed to O‐H stretching vibration, and the band at 2933/cm was attributed to C‐H stretching vibration. The absorption peaks at 1655/cm were assigned to the stretching vibrations C=O, indicating the presence of uronic acid, which was associated with the monosaccharide components (Fu et al., 2020). The peak near 1540/cm is the absorption peak of C‐OH bending vibration. A characteristic absorption peak of 930 and 1078/cm may suggest the presence of β‐glycosidic linkage. Simultaneously, these results indicated that the structure of PEP‐1 and PEP‐2 displayed typical skeletons of polysaccharides.

### 3.4 SEM analysis

**FIGURE 4:**
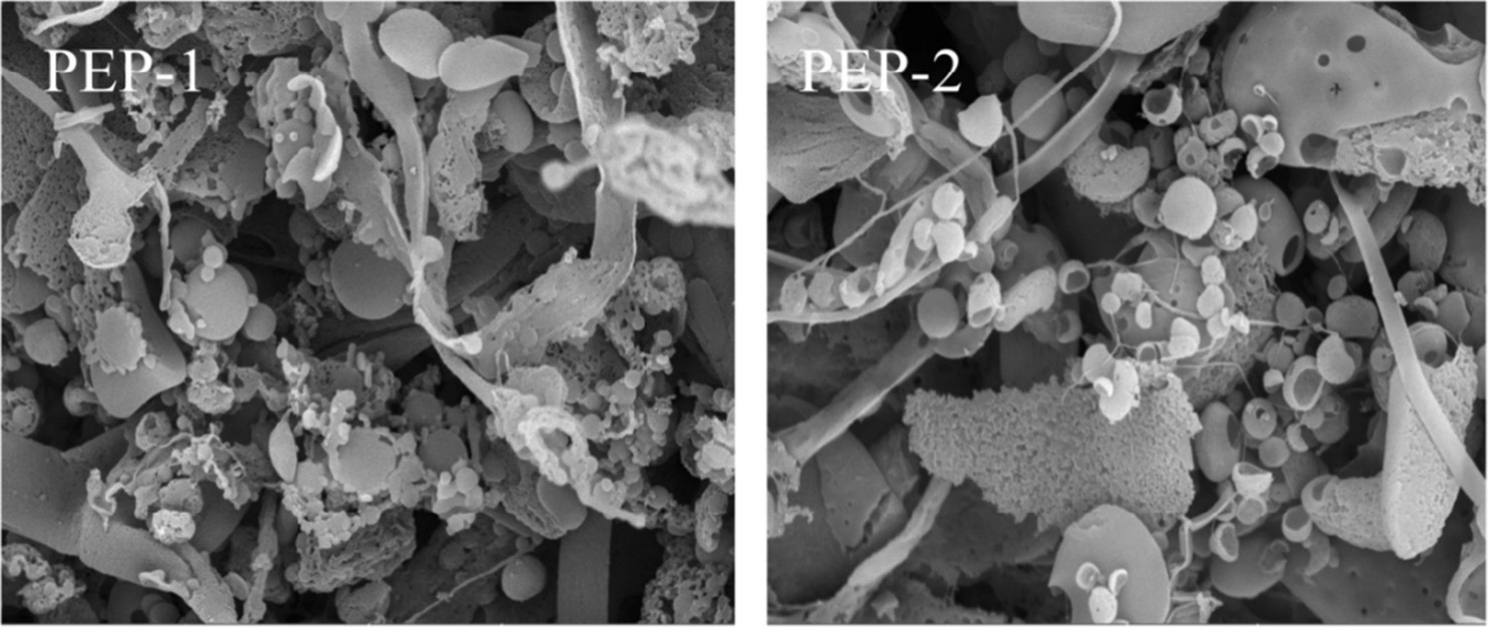
SEM images of the purified polysaccharides from *Phascolosoma esculenta* (500×).

SEM is a straightforward approach to investigating polysaccharides morphology. As shown in Figure 4, there were no significant differences in the apparent forms of PEP‐1 and PEP‐2. PEP‐1 and PEP‐2 exhibited mainly schistose and claviform shapes with intertwining structures. Additionally, it also displayed ellipsoidal particles with smooth surfaces and numerous ostioles.

### 3.5 In vitro antioxidant activity assays

**FIGURE 5:**
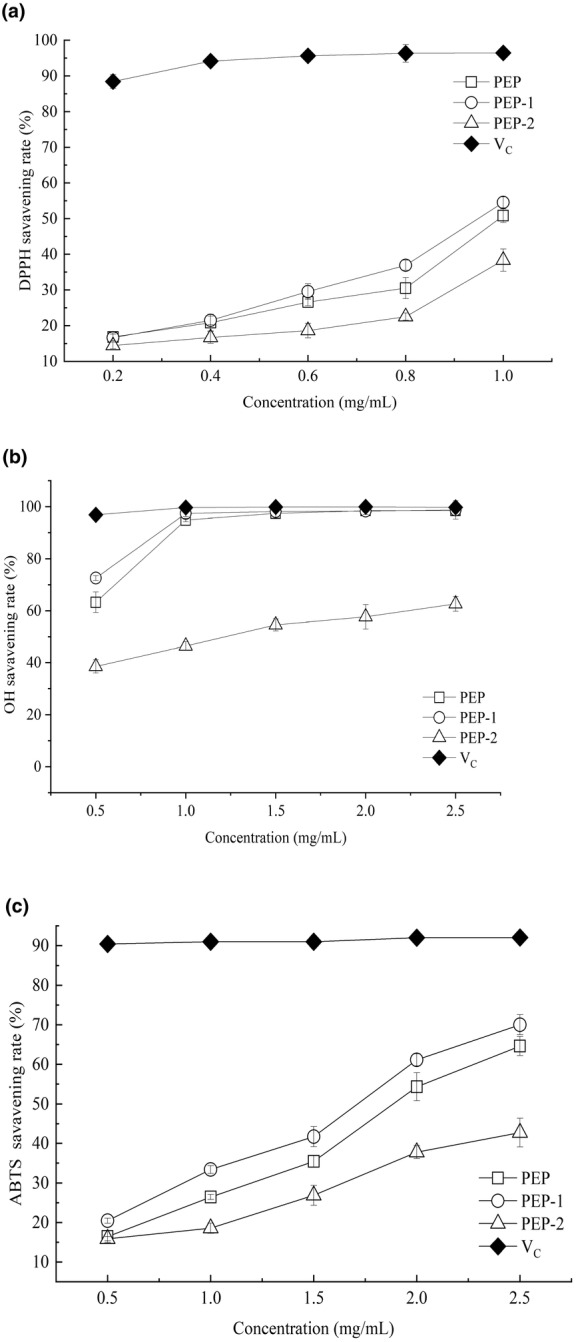
Antioxidant activities of the polysaccharides from *Phascolosoma esculenta*. (a) DPPH radical scavenging rate, (b) •OH radical scavenging rate, and (c) ABTS radical scavenging rate.

The in vitro antioxidant activities of PEP, PEP‐1, and PEP‐2 were investigated in this research. The scavenging activity against radicals of ABTS, •OH, and DPPH were evaluated. Figure 5a illustrated that polysaccharides from *P. esulenta* exhibited a behavior that was dose‐dependent in their capacity to scavenge DPPH. At a dosage of 1.0 mg/mL, DPPH scavenging rates of PEP, PEP‐1, PEP‐2, and Vc were 50.83%, 54.54%, 38.34%, and 96.44%, respectively. Notably, PEP‐1 demonstrated the most effective DPPH scavenging ability compared to the PEP and PEP‐2. While the polysaccharides displayed certain activity in scavenging •OH, their scavenging rates were lower than that of Vc. At a dosage of 2.5 mg/mL, the •OH inhibition rates of PEP, PEP‐1, PEP‐2, and Vc were 98.46%, 98.75%, 62.64%, and 99.76%, respectively (Figure 5b). As presented in Figure 5c, the polysaccharides from *P. esulenta* exhibited scavenging activities against ABTS radicals. At the same concentration, the PEP‐1 had the highest ABTS radicals scavenging rate compared to PEP and PEP‐2, and the PEP‐2 had the lowest ABTS radicals scavenging rate. PEP‐1 had better in vitro antioxidant activity than PEP and PEP‐2. Therefore, PEP‐1 was used for the in vivo antioxidant activity assays.

### 3.6 In vivo antioxidant ability

#### 3.6.1 Effect of polysaccharides on the survival of *C. elegans*

**FIGURE 6:**
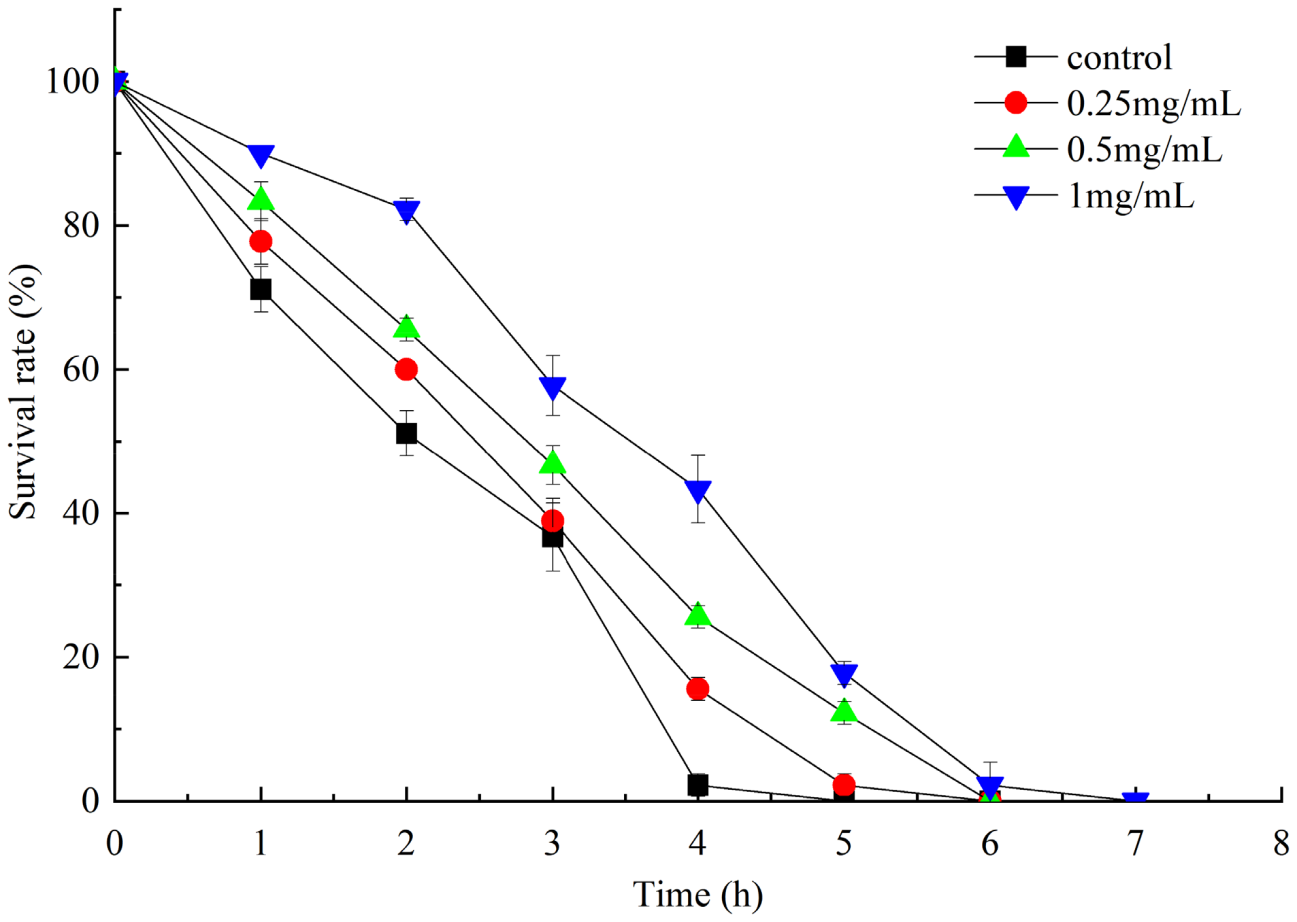
Survival curve of *Caenorhabditis elegans*.

*Caenorhabditis elegans*. survival rate was shown in Figure 6. In comparison to the control group, the survival rate of *C. elegans* treated with PEP‐1 increased by 13.01%, 25.67%, and 38.33% at dosages of 0.25, 0.5, and 1 mg/mL, respectively. *C. elegans* survival rate exhibited a dose–response relationship with the polysaccharides concentration. Polysaccharides of *P. esculenta* exhibited protective effects against oxidative damage to *C. elegans* induced by H_2_O_2_.

#### 3.6.2 Effect of PEP‐1 on ROS level

**FIGURE 7:**
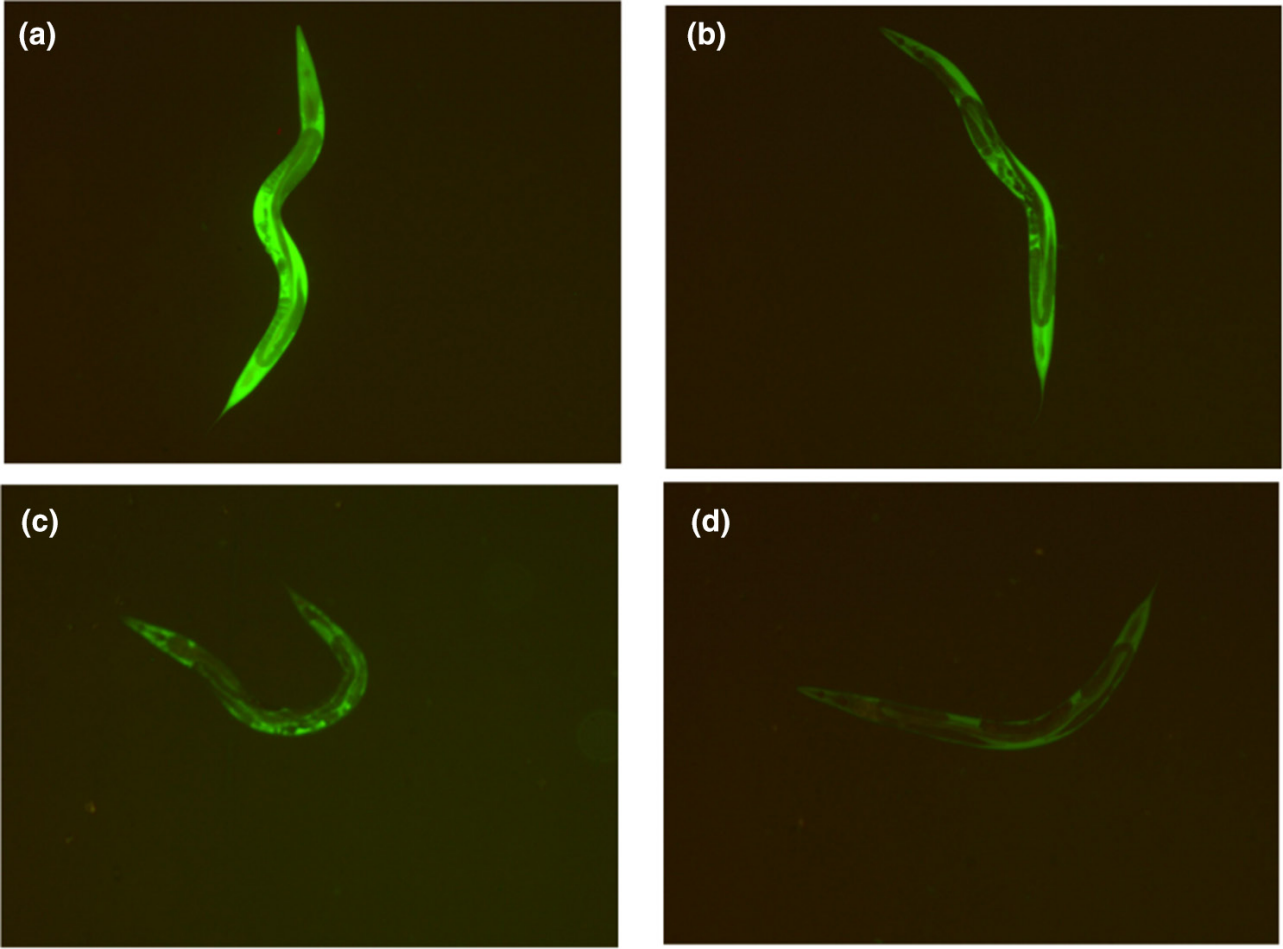
Immunofluorescence staining of ROS in *Caenorhabditis elegans*. (a) 0 mg/mL of PEP‐1, (b) 0.25 mg/mL of PEP‐1, (c) 0.5 mg/mL of PEP‐1, and (d) 1 mg/mL of PEP‐1.

**FIGURE 8:**
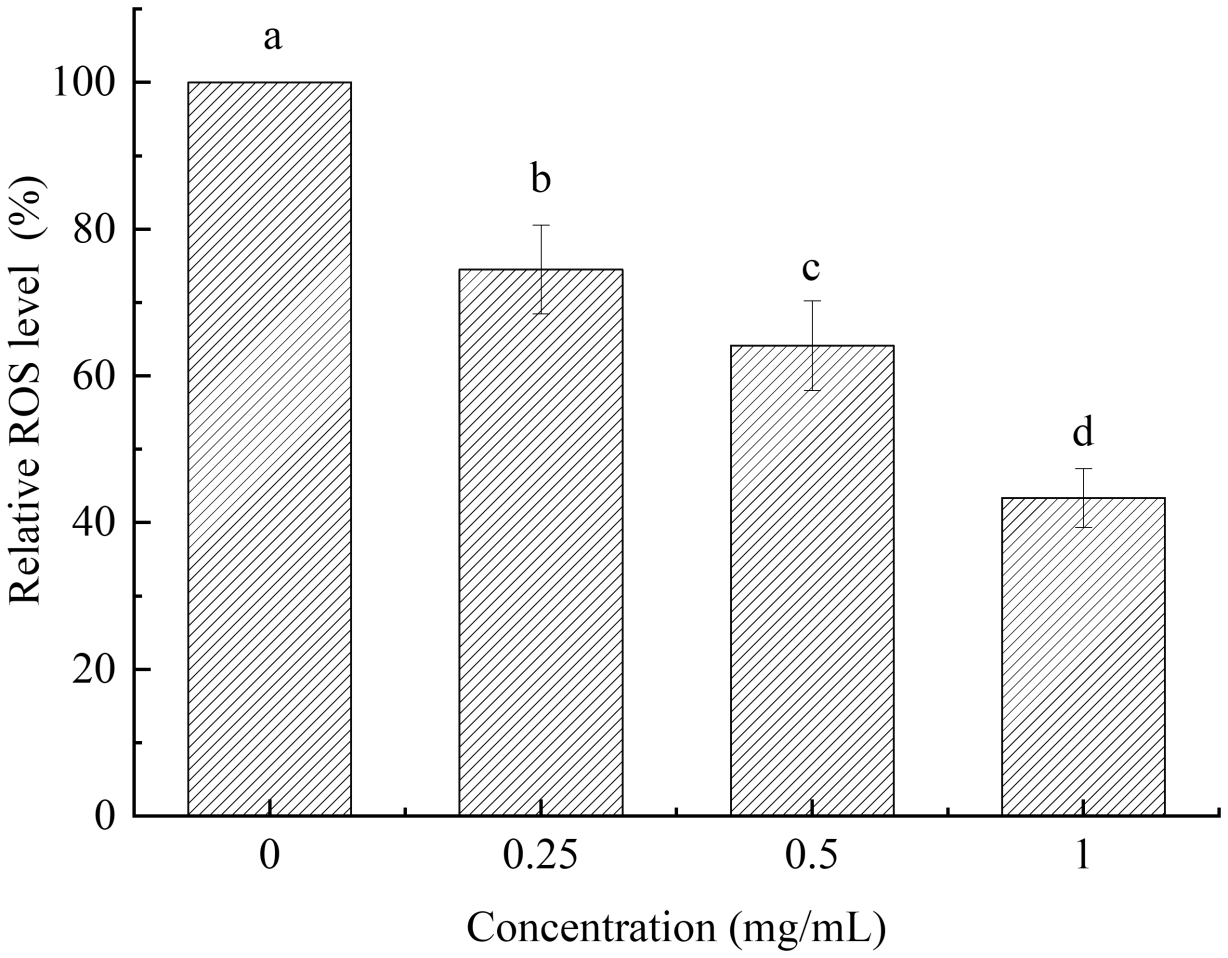
ROS level of *Caenorhabditis elegans*. Values marked by different letters are significantly different (*p* < .05).

Figure 7 illustrated that the *C. elegans* in control group exhibited strong fluorescence intensity. It might suggest that the *C. elegans* were in a situation of oxidative stress. Compared to control group, the PEP‐1 could decrease the fluorescence intensity of *C. elegans*. The fluorescence intensity of *C. elegans* decreased with higher contraction of PEP‐1. *C. elegans* treated with 1 mg/mL PEP‐1 had the lowest fluorescence intensity. There are significant variations among different contractions of PEP‐1 on ROS level in *C. elegans* (*p* <  .05, Figure 8). The increase in contraction of PEP‐1 from 0.25 to 1 mg/mL consequently caused a significant decrease of the ROS level in *C. elegans* (*p* <  .05). At the contraction of 1 mg/mL, PEP‐1 could significantly decrease the ROS level in *C. elegans*. The results of ROS content match with the fluorescence intensity results. These findings indicated that PEP‐1 has the promising potential to inhibit intracellular ROS production in *C. elegans*, alleviating cellular oxidant stress.

#### 3.6.3 Effect of PEP‐1 on fat accumulation in *C. elegans*

**FIGURE 9:**
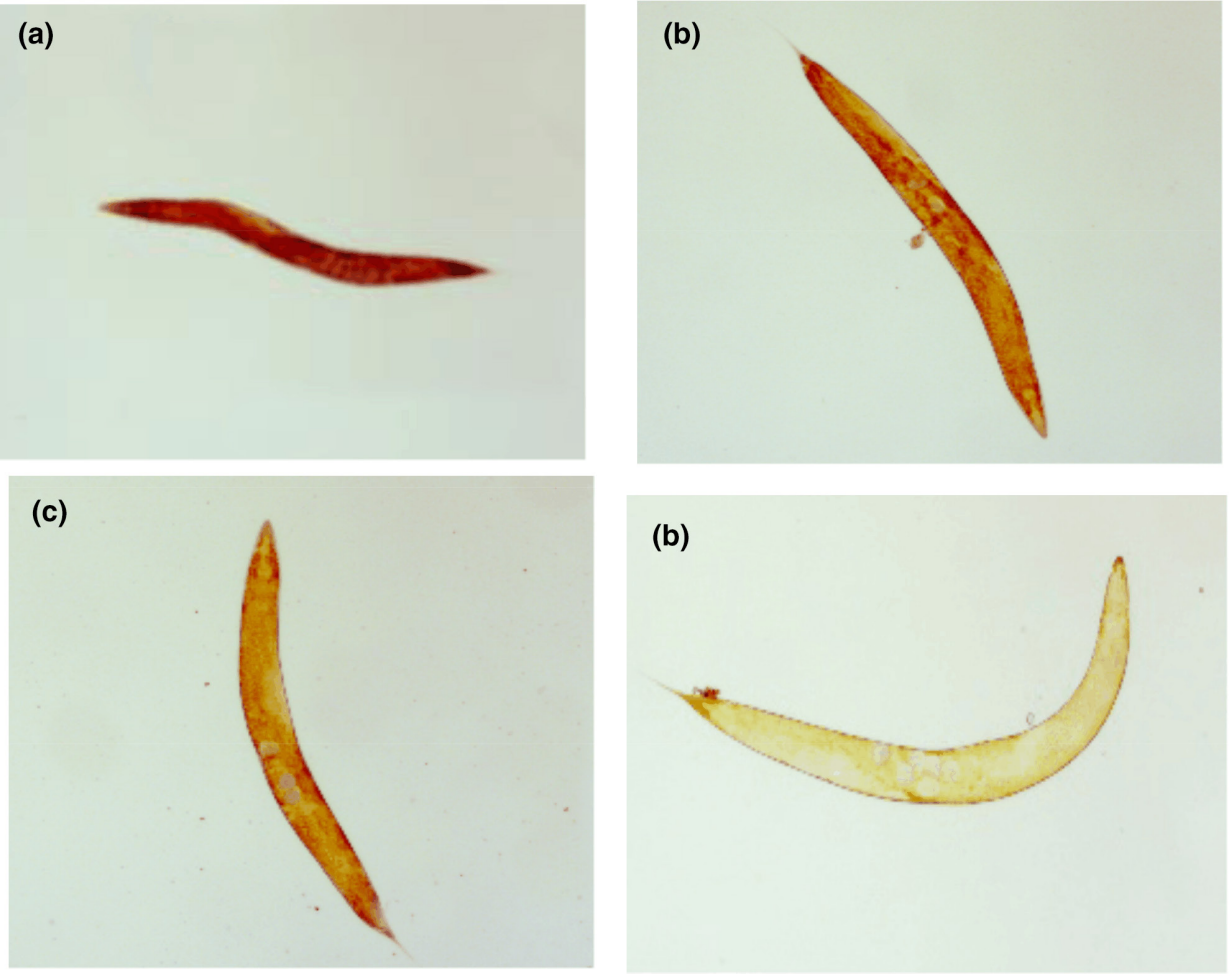
Oil red O staining of *Caenorhabditis elegans*. (a) 0 mg/mL of PEP‐1, (b) 0.25 mg/mL of PEP‐1, (c) 0.5 mg/mL of PEP‐1, and (d) 1 mg/mL of PEP‐1.

**FIGURE 10:**
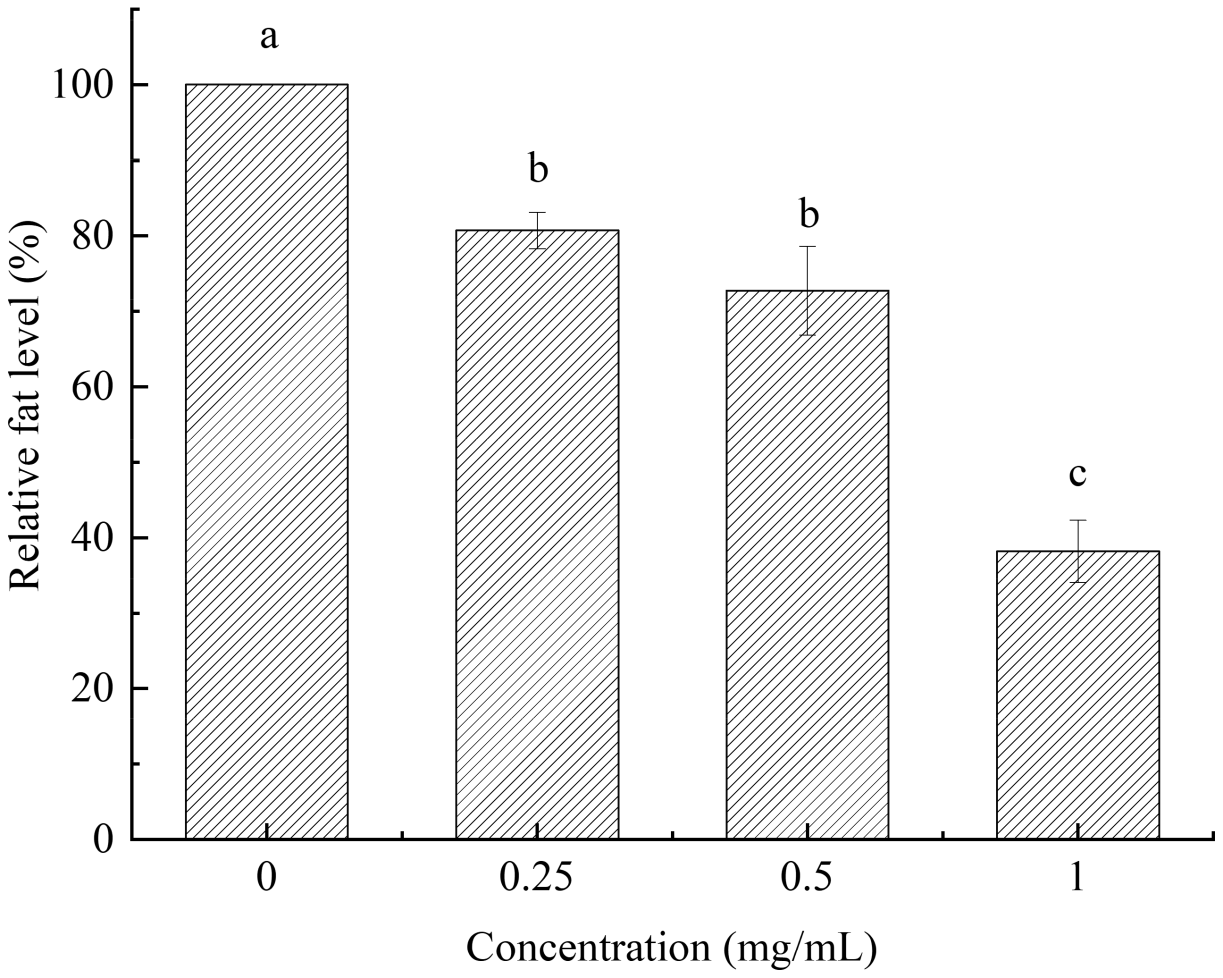
Relative fat level of *Caenorhabditis elegans*. Values marked by different letters are significantly different (*p* < .05).

Figure 9 displayed the color variations of *C. elegans*. The dark color indicated the high‐fat accumulation in *C. elegans. C. elegans* exhibited an orange‐red color in control group, which indicated that the fat content in the control group was at a high level. Subsequently, the increasing contraction of PEP‐1 revealed the color transitioned from saffron yellow to faint yellow, which suggested that the PEP‐1 would decrease the fat accumulation in *C. elegans*. The fat level was significantly influenced in *C. elegans* treated with PEP‐1 (*p* <  .05, Figure 10). As shown in Figure 10, the PEP‐1 had lower fat level at higher concentrations. These results indicated that PEP‐1 has a notable effect in significantly reducing the intracellular fat content of *C. elegans*.

#### 3.6.4 Effect of PEP‐1 on antioxidant enzymes and MDA content

**FIGURE 11:**
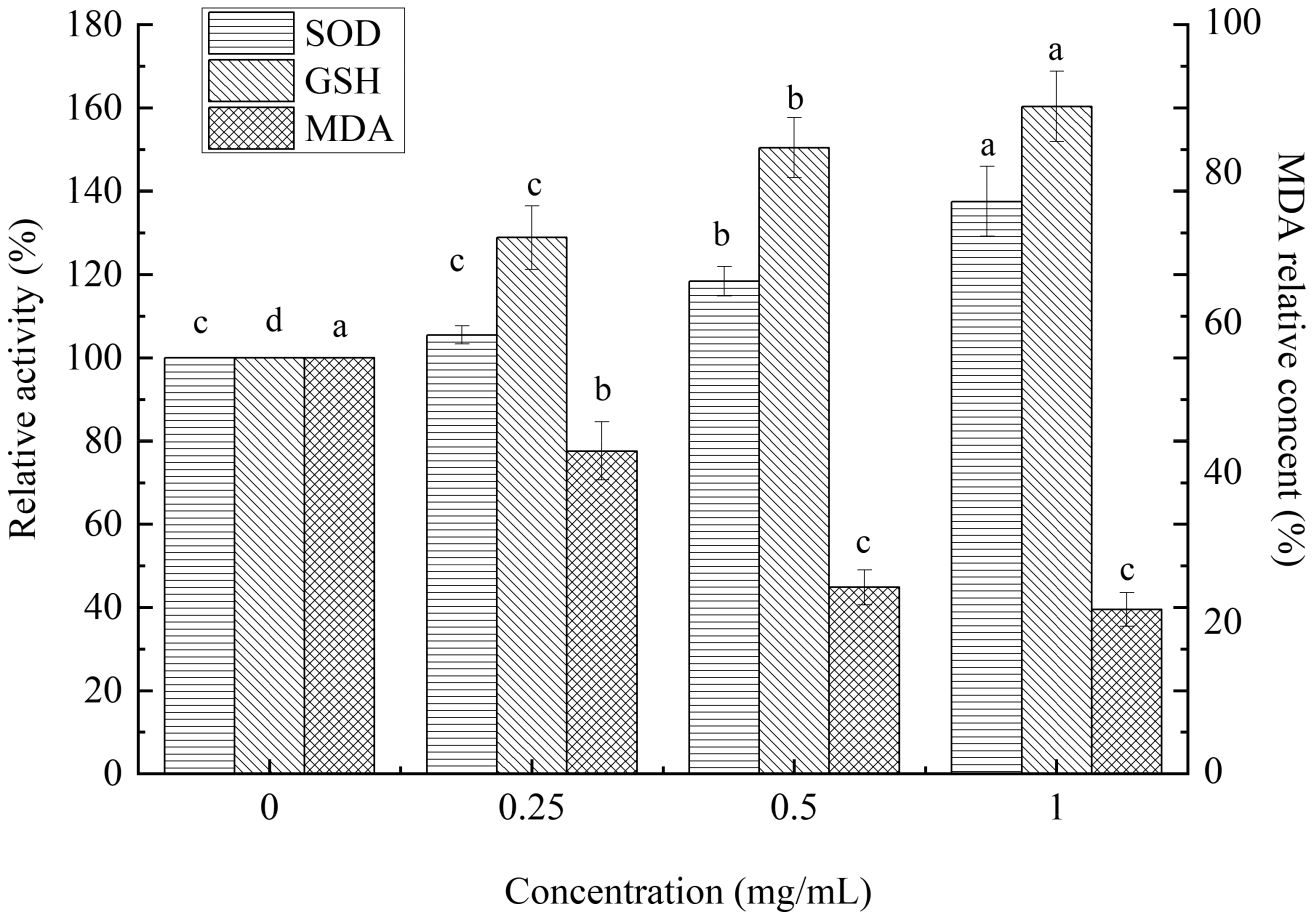
The antioxidant enzymes activities and MDA content. Values marked by different letters are significantly different (*p* < .05).

GSH‐Px and SOD are the primary antioxidant enzymes in *C. elegans*, and responsible for scavenging free radicals and promoting the cell's antioxidant defense system. As presented in Figure 11, GSH‐Px activity in *C. elegans* increased with higher concentrations of PEP‐1. In contrast to the control group, PEP‐1 could improve the activity of GSH‐Px in *C. elegans*. At concentration of 1 mg/mL, PEP‐1 has considerably greater SOD activity than that of 0.25 and 0.5 mg/mL (*p* < .05). As the concentrations of PEP‐1 were increased, the SOD activity also increased. The increase in the concentration of PEP‐1 from 0.25 to 1 mg/mL consequently caused a rapid decrease in MDA content in *C. elegans*. Moreover, compared to control group, the MDA content was significantly reduced in *C. elegans* treated with PEP‐1 (*p* < .05). This research imply that PEP‐1 could enhance antioxidant enzyme activity in *C. elegans*.

## 4 DISCUSSION

The enzyme has been applied to deproteinate the polysaccharide substances. Polysaccharides from marine resources such as abalone and sea cucumber are frequently extracted using papain (Guo et al., 2020; Zhang et al., 2011). Therefore, papain was used to obtain polysaccharides from *P. esculenta*. Presently, ABTS, •OH, and DPPH assays are frequently used to evaluate an antioxidant's potential (Wang et al., 2018; Zhang et al., 2016). In this research, two purified polysaccharides (PEP‐1 and PEP‐2) from *P. esculenta* were harvested. The in vitro antioxidant activity results demonstrated that PEP, PEP‐1, and PEP‐2 had scavenging activities against ABTS, •OH, and DPPH radicals. The free radical scavenging ability of polysaccharides was intimately related to their chemical structures, including the position and type of glycosidic bonds, monosaccharide conformation, and molecular weight (Ferreira et al., 2015; Ji et al., 2017; Wang et al., 2019). In this research, PEP‐1 and PEP‐2 both contained 12 monosaccharides. In comparison to PEP, the PEP‐1 displayed better antioxidant activities. Low molecular weight of polysaccharides would exhibit stronger free radicals scavenging activity (Wang et al., 2019). PEP‐1, with a low molecular weight, demonstrated higher antioxidant activity than PEP‐2. Similar phenomena were found by Shao et al. (2023). PEP‐1, with a high galactose content, exhibited superior antioxidant activity compared to PEP‐2, aligning with Sun's findings that polysaccharides rich in galactose possess enhanced free radical scavenging ability (Sun et al., 2020). Furthermore, some studies suggested that the β‐glycosidic bond configuration contributed to the antioxidant ability of polysaccharides (Song et al., 2018; Wang et al., 2018).

ROS homeostasis is critical for recovery from oxidant stress. Excessive ROS can cause oxidative damage to organisms, reflecting the level of oxidation within cells indirectly (Mekheimer et al., 2012). Natural antioxidants have the potential to alleviate oxidative stress caused by excessive ROS because they have no negative effects. Polysaccharides are excellent sources of natural antioxidants. The CAT, SOD, and GSH‐Px could inhibit ROS accumulation (Ayala et al., 2014). Based on the preliminary results, PEP‐1 had the strongest in vitro antioxidant activity, thus PEP‐1 was selected for further assessment in vivo study. The in vivo antioxidant activity of PEP‐1 was examined using the *C. elegans* oxidative stress resistance assay. The results showed that PEP‐1 exhibited protective effects against oxidative damage to *C. elegans* induced by H_2_O_2_. Compared to the control group, PEP‐1 could improve the SOD and GSH‐Px activities, and decrease the ROS and MDA content of *C. elegans*. Therefore, the PEP‐1 exhibited in vivo antioxidant activity.

## 5 CONCLUSION

The polysaccharides from *P. esculenta* and its purified polysaccharides (PEP‐1 and PEP‐2) were prepared. There were 12 monosaccharides in PEP‐1 and PEP‐2. Furthermore, the infrared spectra of PEP‐1 and PEP‐2 were comparable, and both had β‐glycosidic bonds. PEP‐1 demonstrated stronger in vitro antioxidant activity than PEP and PEP‐2 due to its lower molecular weight. PEP‐1 could increase SOD and GSH‐Px activities while preventing ROS accumulation. The findings demonstrated the potential for polysaccharides from *P. esculenta* to be developed as antioxidant agents in the food industry, thereby expanding the application of polysaccharides from *P. esculenta* as functional foods. Furthermore, the antioxidant mechanism of polysaccharides from *P. esculenta* requires further investigation.

## AUTHOR CONTRIBUTIONS

**Fengfang Zhou:** Resources (lead); validation (lead); writing – original draft (equal). **Binxin Cai:** Data curation (equal); formal analysis (equal). **Shaojiang Ruan:** Investigation (equal); methodology (equal); project administration (equal). **Qi Wei:** Project administration (equal); writing – review and editing (equal).

## CONFLICT OF INTEREST STATEMENT

The authors declare that they do not have any conflict of interest.

## Data Availability

The data that support the findings of this study are available on request from the corresponding author.
